# Models for Predicting Dementia Risk in Low‐ and Middle‐Income Countries

**DOI:** 10.1002/alz.088573

**Published:** 2025-01-09

**Authors:** Maha Alshahrani, Blossom CM Stephan, Mario Siervo, Serena Sabatini, Eugene Yee Hing Tang, Jacob Brain, Aliya Naheed, Eduwin Pakpahan, Louise Robinson, Devi Mohan

**Affiliations:** ^1^ Curtin enAble Institute, Faculty of Population Health, Curtin University, Perth, Western Australia Australia; ^2^ Curtin University, Perth, Western Australia Australia; ^3^ University of Nottingham, Nottingham, Nottinghamshire United Kingdom; ^4^ School of Psychology, University of Surrey, Guildford, Surrey GU2 7XH United Kingdom; ^5^ Newcastle University, Newcastle United Kingdom; ^6^ The University of Adelaide, Adelaide, SA Australia; ^7^ Health Systems and Population Studies Division, icddrb, Mohakhali, Dhaka, 1000 Bangladesh; ^8^ Physics and Electrical Engineering, Northumbria University, Newcastle upon, Tyne United Kingdom; ^9^ Newcastle University, Newcastle upon Tyne United Kingdom; ^10^ School of Public Health, The University of Queensland, Brisbane, QLD Australia; ^11^ Jeffrey Cheah School of Medicine and Health Sciences, Monash University Malaysia, Bandar Sunway Malaysia

## Abstract

**Background:**

Most people with dementia reside in low‐ and middle‐income countries (LMICs) where resources, research, services, and support are often very limited. Research into dementia risk prediction is scarce in LMIC settings, and those prediction models developed in high‐income countries generally do not transport well to LMICs. This suggesting a dire need for LMIC specific dementia risk models.

**Method:**

We synthesised the evidence from our three previous systematic reviews (covering all literature from inception to 2023 from PubMed, Embase, and PsychInfo) on dementia risk prediction modelling. The aim was to identify models that have been specifically developed and tested specifically in LMICs. There were no language or time restrictions applied.

**Result:**

To date, over 50 different dementia risk prediction models have been developed and tested with only 7 models reported from two LMICs including five studies from China and two studies from Mexico. The models incorporated variables typically linked to dementia including demographics (e.g., age, sex, education), health (e.g., diabetes, hypertension, heart disease) and lifestyle (e.g., smoking and alcohol) variables. The 7 models also have varying degrees of predictive accuracy (c‐statistic range 0.65 [95%CI: 0.64–0.67] to 0.92 [95%CI: 0.88‐0.95]) and none has undergone external validation. These models have been developed using traditional statistical approaches including Cox and Logistic Regression. Further, model development has not considered factors such as socioeconomic status, literacy, access to healthcare, diet, stress, pollution, and workplace hazards that may be crucial in predicting dementia risk in LMICs.

**Conclusion:**

There is an urgent need to create context‐specific dementia prediction models to inform the development of risk reduction and preventative interventions in LMICs where dementia case numbers are greatest. Dementia risk model development and testing need to be extended to LMICs across different regions (e.g., Asia, Middle East, Global South, Africa) and income levels (e.g., low, lower‐middle, and upper‐middle income).

**Recommendations:**

Greater investment is needed into understanding dementia, and its risk factors in LMICs to inform the development of risk mitigation programs. Research should focus on developing accurate, resource‐conscious models with affordable and obtainable variables for identifying those individuals likely to benefit the most from interventions targeting risk reduction.

